# The Destructive Effect of Ingroup Competition on Ingroup Favoritism

**DOI:** 10.3389/fpsyg.2018.02207

**Published:** 2018-11-23

**Authors:** Youxia Zuo, Bing Chen, Yufang Zhao

**Affiliations:** ^1^Center for Studies of Education and Psychology of Ethnic Minorities in Southwest China, Southwest University, Chongqing, China; ^2^School of Life Sciences, Southwest University, Chongqing, China

**Keywords:** ingroup favoritism, competitive intragroup outcome interdependence, social identification, intergroup competition, intergroup behavior

## Abstract

Ingroup favoritism has been widely verified in the context of intergroup competition; however, how competition among ingroup members affects ingroup favoritism remains unclear. We hypothesized that competition among ingroup members may disrupt individuals’ ingroup-favoring behavior because of conflicts of interest; we tested this hypothesis in two studies. In Study 1, we manipulated competitive intragroup outcome interdependence (present vs. absent) and the manner in which results were presented (public vs. anonymous). We found that regardless of result presentation, when competitive intragroup outcome interdependence was present, ingroup members did not exhibit ingroup favoritism; when such interdependence was absent, they showed ingroup favoritism. In Study 2, we introduced the manipulation of social identification, and reverified the main result that individuals under competitive intragroup outcome interdependence do not exhibit ingroup favoritism. Even the degree of social identification—a vital factor for intergroup behavior—could not moderate the destructive effect of competitive intragroup outcome interdependence on ingroup favoritism. Together, these findings indicate that ingroup favoritism would indeed be damaged by competition among ingroup members.

## Introduction

Most people will exhibit the systematic tendency to favor members of one’s own group over those of other groups, which could be described as “ingroup favoritism.” There is a plethora of experimental studies verifying that individuals exhibit greater prosocial behavior or favoritism toward ingroup members when compared with outgroup members (Ahmed, 2007; Balliet et al., 2014). A pioneering study of Tajfel et al. (1971) employed an allocation matrix, in which individuals needed to distribute valued points to anonymous ingroup or outgroup members by the series of point allocation scales, and found that subjects favored their own group in the distribution of valued resources between the ingroup and outgroup. A number of other studies replicated and extended the findings about ingroup favoritism using various paradigms. Studies using the prisoner’s dilemma and public-goods dilemma games have shown that individuals usually cooperated more with ingroup members in social dilemmas (Krupp et al., 2008). Moreover, individuals tend to give more resources and trust their own membership group in trust games (Ioannou et al., 2012) and ultimatum games (Mcleish and Oxoby, 2011). However, all these experimental paradigms substantially contain positive outcome interdependence among ingroup members (i.e., the outcomes of ingroup members are positively correlated), which will facilitate ingroup favoritism (Balliet et al., 2014; Everett et al., 2015). In dictator games—without outcome interdependence among ingroup members—ingroup favoritism was not observed, because participants acting as dictators could make a unilateral decision about allocating any amount of money to recipients, and the recipients had no effect on the allocation other than to accept the amount. While some studies using dictator games found that individuals would favor ingroup members, others found no evidence for ingroup favoritism (Liebe and Tutic, 2010; Lei and Vesely, 2010). That is, when outcome interdependence is absent, the tendency of ingroup favoritism is inconsistent.

However, often there is negative outcome interdependence (i.e., the outcomes of ingroup members are negatively correlated) in human groups. A typical negative outcome interdependence within the group is competitive intragroup outcome interdependence. It happens when group members perceive their goals as competitively linked with those of other members (i.e., intragroup competition; De Dreu, 2007). In most organizations, apart from intragroup cooperation, intragroup competition is another critical management strategy to motivate employees, promote team performance, and increase the organization’s market share (Tjosvold, 1998). It is common for individuals to compete with their ingroup members for promotions, raises, and praise from supervisors (Rees and Segal, 1984; Birkinshaw, 2002). Yet, intragroup competition can also result in negative outcomes, including high levels of employee anxiety, resource wastage, and narrowed range of attention (Birkinshaw, 2002; Naidoo, 2013), and can interfere with solidarity, cohesion, and commitment (Leventhal, 1976). At present, it is unclear whether individuals perceiving competitive intragroup outcome interdependence would favor ingroup members; this deserves exploration.

Although competitive intragroup outcome interdependence maybe a valid way of encouraging group members to improve performance, it may reduce individuals tendency to favor the ingroup in discriminatory behavior and decision-making (Tjosvold, 1998). Higher competitive intragroup outcome interdependence was accompanied by a decline in productivity and cooperation (Brown, 2000). Moreover, competitive intragroup outcome interdependence lead to more knowledge-hiding among employees (Bavik, 2015), decline of interpersonal attraction among members (Rees and Segal, 1984), and even in-fighting (Birkinshaw, 2002). Based on previous research, competitive intragroup outcome interdependence would logically prevent ingroup favoritism, as it suppresses ingroup harmony.

According to the theory of bounded generalized reciprocity (BGR), ingroup favoritism is the result of interdependence and is motived by reciprocal expectations toward ingroup members (Rabbie et al., 1989; Yamagishi et al., 1999). BGR assumes that human groups provide a container for a generalized exchange network in which individuals exhibiting prosocial behavior are likely to be paid back both directly and indirectly (i.e., by building a good reputation through prosocial behavior, one can later obtain favor from others; Seinen and Schram, 2000; Nowak and Sigmund, 2005). When reciprocal expectations are absent, ingroup favoritism is weakened, and may even disappear (Everett et al., 2015). Individuals showed ingroup-favoring allocation decisions only when other members showed similar decisions; and if their favorable allocations to members would not determine their own payoff, they did not exhibit ingroup favoritism (Yamagishi and Kiyonari, 2000). Thus, we can infer that interaction with ingroup members functions as a heuristic of reciprocal expectations within the group, while competitive intragroup outcome interdependence could erase this heuristic. Therefore, individuals may not favor their ingroup members under intragroup competition.

Social identification may act as a buffer for the damage on ingroup favoritism through competitive intragroup outcome interdependence. According to the social identity theory (SIT), once people have identified with a certain group, they seek to develop and maintain a positive self-concept through maximizing the positive distinctiveness of their ingroup, in contrast to an outgroup (Tajfel and Turner, 1979). The degree of identification with a certain group seems to guide individuals’ perceptions and behaviors. Stronger group identification is associated with a greater likelihood of categorizing oneself as an ingroup member (Spears et al., 1997), remaining committed to the ingroup in the face of a threat (Ellemers et al., 1996). It also tends to make people exhibit more prosocial behavior toward ingroup members (Cremer and Vugt, 2002). Moreover, people who identify strongly with their own group tend to exhibit greater ingroup favoritism than do those who identify less strongly with their group (Leonardelli and Brewer, 2001). That is to say, social identification significantly and actively induces ingroup favoritism. Thus, we infer that social identification may moderate the destructive effect of competitive intragroup outcome interdependence on ingroup favoritism.

The minimal group paradigm was developed by Tajfel et al. (1971) to study the minimal necessary and sufficient conditions of intergroup discrimination. In this paradigm, participants are classified into two arbitrary groups on the basis of trivial or explicitly random criteria. There is no previous history of relations and social interaction within or between group members. Participants need to indicate their preferred decisions about the distribution of financial reward between anonymous ingroup and outgroup members. Social-psychological studies have already shown that the random categorization of people in two groups is sufficient to induce intergroup discrimination (Stroebe et al., 2005). Consequently, to verify our hypotheses, we conducted two experiments using Tajfel’s minimal-group paradigm, wherein we examined whether competitive intragroup outcome interdependence would cause ingroup favoritism to collapse. Additionally, Fehr and Fischbacher (2004) proposed that people act in a prosocial manner toward group members in order to build their reputation, and because they perceive such prosociality to be socially approved and understand the costs of violating this social norm. Selfish behavior among ingroup members also might be punished more than that among outgroup members (Mendoza et al., 2014). In multiple-person social dilemmas, agents can hide their actions behind the veil of anonymity, so the cost one can impose on those who fail to cooperate are diffused and diluted, thus having less threatening impact (Isaac and Walker, 1988). Consequently, in Study 1, we manipulated the competitive intragroup outcome interdependence (present vs. absent) and simultaneously performed a manipulation of the manner of presentation of the allocation results (public vs. anonymous) to determine individuals’ ingroup-favoring behavior across different allocation conditions of Tajfel’s paradigm. In Study 2, we re-verified the effect of competitive intragroup outcome interdependence on ingroup favoritism when introducing identification manipulation. Especially, we would clarify whether the degree of group identification could moderate the effect of competitive intragroup outcome interdependence on ingroup favoritism.

## Study 1

This study sought to provide initial empirical evidence that competitive intragroup outcome interdependence negatively influences ingroup favoritism in the minimal group paradigm (Tajfel, 1974). We used Tajfel’s allocation matrices to indicate participants’ preferences for the ingroup or outgroup.

### Methods

#### Participants and Design

This experiment comprised a 2 (Competitive Intragroup Outcome Interdependence: present vs. absent) × 2 (Results Presentation: public vs. anonymous) mixed design. Results presentation was a within-subjects factor, while competitive intragroup outcome interdependence was a between-subjects factor. We recruited a total of 139 participants (95 women; *M*_age_ = 20.97, *SD* = 2.31) and then randomly assigned them to two competitive intragroup outcome interdependence conditions (present, *n* = 74; absent, *n* = 65). Sample size was determined, based on a mixed design ANOVA, by using G^∗^Power 3.1.9.2. With a hypothesized medium-sized effect of 0.25, alpha of 0.05, power of 0.9, two groups, and two measurement occasions, the estimated sample size was 130. To ensure robustness, we required a total sample size of 139. This study has been approved by the Ethics Committee of Southwest University and written informed consent has been obtained from each participant, who received remuneration for participating in this study.

#### Procedure

##### Grouping

In line with the minimal group paradigm procedure, we invited 6 participants, with no history of interaction with each other before the experiment, to take part in the experiment at a fixed time (if participants missed the experiment, we used assistants to replace them to avoid disrupting the experiment). After the participants took their seats in six separate cubicles, they were informed that the study would be related to their perception style (i.e., underestimation vs. overestimation) and daily behavior. They were asked to estimate the number of dots on ten pictures, and then record their estimates on sheets of paper. After this task, we ostensibly categorized participants into underestimator (a_1,_ a_2,_ and a_3_) and overestimator (b_1,_ b_2,_ and b_3_) groups according to their estimates; in fact, participants were averagely and randomly categorized. Therefore, each group contained three participants.

##### Competitive intragroup outcome interdependence

Following the grouping, underestimator and overestimator groups were categorized based on the experimental conditions (presence or absence of competitive intragroup outcome interdependence). The presence or absence of competitive intragroup outcome interdependence relied on a reward mechanism. The participants were asked to complete tasks to obtain points, which would be used in determining their additional reward. In the “present” condition (where competitive intragroup outcome interdependence was present), participants were told that they would compete for reward within the group. Specifically, the person who had the highest points in each group would obtain an additional 10 yuan (i.e., 1.51 U.S. dollars) as a reward apart from the remuneration they were already receiving for participating in the experiment; the others in the group would receive only the basic remuneration. In the “absent” condition (where competitive intragroup outcome interdependence was not present), participants were told that anyone whose points exceeded 106 (the median) would be awarded an additional 10 yuan. Namely, participants did not need to compete with others within the group.

##### The rule for obtaining points

All participants were told that they needed to complete two tasks (tasks I and II), but all of them would receive points for only one of the two, which was determined by drawing lots. Moreover, participants would get information that in each group, one (e.g., a_1;_ b_1_) of the three members could obtain points from task I and that the other two (a_2_ and a_3;_ b_2_ and b_3_) could gain points from task II. Factually, we manipulated the drawing of the lots to ensure that all participants could only obtain points from task II. The reason for this manipulation was the differing purpose of the two tasks.

##### Task I and II

Task I aimed to identify participants’ preference for the ingroup or outgroup in two competitive intragroup outcome interdependence conditions. Specifically, Task I was a points allocation task, wherein participants had to indicate their preferred allocation between an anonymous underestimator (a_1_) and an anonymous overestimator (b_1_) (i.e., in the participants’ minds, between an ingroup and an outgroup member who would obtain points from task I). In order to hide our manipulation of drawing lots, the experimenter intentionally emphasized that the allocation of a_1_ and b_1_ would not be calculated when determining their own final points. Task II was designed to eliminate reciprocal interdependence between participants to make them truly exhibit preference for either the ingroup or outgroup members in task I. What’s more, Task II could also be used as the basis for awarding because it authentically determined participants’ final points. Task II was similar to drawing lots, in that each participant randomly chose a scrip containing the points they could obtain. There were 46 scrips in total, each containing a number between 60 and 152 (half < 106). As such, participants’ obtained points from task II were luck-based. Consequently, all participants would realize that their final points and acquirable reward actually depended on their luck, which eliminated reciprocal interdependence. After the participants completed the two tasks, the experimenter calculated and ranked each participant’s points obtained from task II to determine their reward. Subsequently, the experimenter explained the intended experimental design and the basis for determining the reward.

##### Results presentation

**FIGURE 1:**
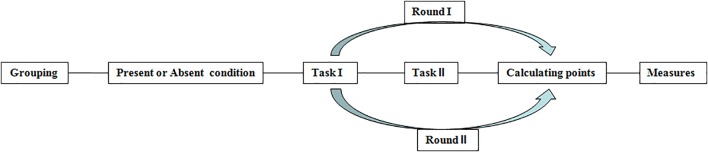
The procedure of study 1.

The experiment took place in two rounds; participants needed to complete task? first, and then task II in each round (see Figure 1). Simultaneously, we performed a within-subjects manipulation of how the results of the tasks were presented. In the first round, before conducting task I and II, the experimenter told participants that the results presentation of that round was anonymous, i.e., they simply calculated everyone’s final points in secret and did not announce details about the distribution of each participant’s points. However, for the second round, the participants were told that the results presentation of that round was public before they completed the two tasks. Specifically, the experimenter publically announced who could obtain points from task I or II and how each member distributed points between the overestimator and underestimator after the two tasks had been completed. The reason for conducting the anonymous results presentation first was to avoid imitation and learning after the public round. After the two rounds, we gave participants their final remuneration.

#### Measures

##### Perceived competitive intragroup outcome dependence

To confirm the competitive intragroup outcome interdependence manipulation, participants responded to two items on a 7-point response scale (1 = *strongly disagree* to 7 = *strongly agree*) from Stroebe et al. (2005). One of the items directly assessed perceived competition with ingroup members: “During the allocation task, I realized that I was competing with members of the overestimator group or underestimator group (matching to one’s ingroup) to obtain the award.” The other item directly assessed was the perceived reciprocal interdependence with other participants: “During the allocation task, I realized that I was strongly dependent on other participants to obtain the award.”

##### Allocation matrices

In task I, participants were asked to indicate their preference for the allocation of points between an ingroup and an outgroup member using eight matrices adapted from Tajfel et al. (1971). Difference scores were calculated as indexes of intergroup discrimination, which were calculated by subtracting outgroup members’ allocations from ingroup members’ allocations across the eight matrices (Diehl, 1988). Positive difference scores indicated ingroup favoritism, and negative difference scores indicated outgroup favoritism. Difference scores values near zero indicated a tendency toward fairness. Moreover, some matrices could also reveal allocation strategies based on pull scores. We focused on the strategies of Allocation Matrix B (see Table 1; Tajfel et al., 1971, Experiment 2), which involved and compared the two main strategies pertaining to intergroup discrimination. Matrix Type B produced the following two pull scores: one indicated the maximum differentiation (MD) strategy and the other indicated the maximizing ingroup profit (MIP) and/or maximizing joint profit (MJP) strategy (this matrix cannot distinguish between MIP and MJP). In matrix-type B, where allocations to the group O are in the top row, a predominance of response by Group O members toward the right extreme suggests that subjects employ the MIP/MJP strategy. Choices toward the left extreme of the same matrix are indicative of the influence of maximum differentiation (MD). Pull scores, reflecting the different discrimination and strength of strategy, ranged from -12 to 12. Positive (negative) pull scores for MD indicated a concern for maximizing differentials between ingroup and outgroup allocations in favor of the ingroup (outgroup), while the positive (negative) pull scores for MIP/MJP indicated choices for maximizing the award for the ingroup (outgroup) and/or maximizing (minimizing) the outcomes for both groups combined. For the methods of calculating the pull scores we referred to Bourhis et al. (1994).

**Table 1:** Matrix Types B: MD vs. MIP/MJP, the allocation from the view of a member of Group O.

Points to Member S of Group O	7	8	9	10	11	12	13	14	15	16	17	18	19
Points to Member P of Group U	1	3	5	7	9	11	13	15	17	19	21	23	25
Choice	√												√

### Results

#### Manipulation Checks

All participants’ results were analyzed. The scores of perceived intragroup competition of the two conditions were submitted to independent-samples *t*-test, and the result revealed that the participants in the present condition (*M* = 4.88, *SD* = 1.6) perceived a higher sense of competition than did those in the absent condition (*M* = 3.51, *SD* = 1.63), *t*(137) = 2.93, *p* < 0.001, *d* = 0.86. However, there was no significant difference between the two conditions in terms of the perceived reciprocal interdependence with other participants. Further, we wanted to check whether participants in the present condition really perceived intragroup competition rather than just perceiving a stronger intragroup competition compared to the participants in the absent condition; while participants in the absent condition did not perceive intragroup competition rather than just perceiving less sense of intragroup competition compared to the participants in the present condition. So, we also conducted a comparison of the scores of perceived intragroup competition to 4 (mid-value) using one-sample *t*-test, if the scores were significantly higher than 4, suggesting a perception of intragroup competition; if that lower than 4, suggesting an absent feeling of intragroup competition. The results revealed that members in the present condition had scores that were significantly higher than 4 (*M* = 4.88, *SD* = 1.6), *t*(73) = 4.71, *p* < 0.001, *d* = 0.55. In contrast, participants in the absent condition had scores that were significantly lower than 4 (*M* = 3.51, *SD* = 1.63), *t*(64) = -2.43, *p* = 0.018, *d* = 0.30. Namely, the manipulation of competitive intragroup outcome interdependence succeeded; participants in the present condition really perceived intragroup competition while those in the absent condition did not. Similarly, we tested whether there was a difference in perceived reciprocal interdependence between the two conditions. The independent-samples *t*-test did not suggest a significant difference between the present and absent conditions. Further, we also checked whether participants in each condition really did not perceive reciprocal interdependence with other participants. The one-sample *t*-tests revealed that all participants had scores that were significantly lower than 4 for perceived reciprocal interdependence with other participants: for the present condition, *M* = 3.12, *SD* = 1.74, *t*(73) = -4.35, *p* = 0.001, *d* = 0.51, and for the absent condition, *M* = 2.62, *SD* = 1.38, *t*(64) = -8.11, *p* = 0.001, *d* = 1, indicating that we controlled the sense of reciprocal interdependence among participants.

#### Ingroup Favoritism

Considering that the participants were placed in small groups, and that independence of observations within groups is a key assumption of the *t*-test and ANOVA, we computed intra-class correlation coefficients using the procedure suggested by Snijders and Bosker (1999), to test whether observations within groups were independent. The results confirmed that the intra-class correlation coefficients were trending to 0 regardless of targets (DS, MD or MIP/MJP), which demonstrated that observations within groups were independent. Therefore, we then analyzed the data using the *t*-test and ANOVA.

Difference scores, used as an index of intergroup discrimination (as shown in Table 2), were included in a 2 (Competitive Intragroup Outcome Interdependence) × 2 (Results Presentation) analysis of variance (ANOVA). The result revealed a significant main effect of competitive intragroup outcome interdependence *F*(1,137) = 20.56, *p* = 0.001, η^2^ = 0.13, but no other main or interaction effects were significant. Inspection of the significant main effect suggested that the difference scores of members in the absent condition (*M* = 20.65, *SD* = 5.48) were greater than were those of members in the present condition (*M* = -13.40, *SD* = 5.14).

**Table 2:** Mean difference scores of members according to competitive outcome interdependence and results presentation.

	Competitive outcome interdependence	
	Present	Absent
Anonymous presentation	-15.45 (54.41)	19.06 (43.78)
Public presentation	-11.35 (49.94)	22.23 (41.92)

*Values are Mean (SD)*.

Further, we subjected the difference scores to one-sample *t*-tests to determine participants’ degree of discrimination within different conditions. In the absent condition, difference scores were greater than zero regardless of whether the results presentation was public, *t*(64) = 4.28, *p* < 0.001, *d* = 0.53 or anonymous, *t*(64) = 3.51, *p* = 0.001, *d* = 0.44, which suggests that there was significant discrimination in favor of the ingroup. In contrast, in the present condition, difference scores were significantly lower than zero when results presentation was anonymous, *t*(73) = -2.44, *p* = 0.017, *d* = 0.28, and marginally but significantly lower than zero when the presentation was public, *t*(73) = -1.96, *p* = 0.054, *d* = 0.23, which suggests an outgroup-favoring discrimination.

#### Allocation Strategies

Pull scores were calculated to illustrate the relative strengths of the different discrimination strategies. We referred to Bourhis et al. (1994) when analyzing the pull scores. First, we determined whether participants had actually employed any of these pulls by testing whether the pull scores obtained in each condition were significantly different from zero. Then, we analyzed the differing use of these pulls across the conditions using an ANOVA. The two pull scores were subjected to one-sample *t*-tests. The results indicated that both MD and MIP/MJP pull scores were significantly different from zero, indicating that they had actually been used by participants.

The result for the MIP/MJP pull indicated that in the present condition, participants showed a significant motivation for maximizing outgroup profit and/or maximizing joint profit, regardless of whether the results presentation was public (*M* = -3.14, *SD* = 6.44), *t*(73) = -4.19, *p* < 0.001, *d* = 0.49, or anonymous (*M* = -15.45, *SD* = 6.84), *t*(73) = -2.72, *p* = 0.008, *d* = 2.26; in contrast, participants in the absent condition exhibited a strategy of MIP/MJP, again regardless of whether the results presentation was public (*M* = 2.80, *SD* = 7.17), *t*(64) = 3.15, *p* = 0.002, *d* = 0.39 or anonymous (*M* = 3.43, *SD* = 7.60), *t*(64) = 3.64, *p* = 0.001, *d* = 0.45. For the MD pull, participants in the absent condition preferred maximizing the relative difference in favor of the ingroup at the cost of sacrificing the maximum profit of the ingroup, regardless of a public (*M* = 1.45, *SD* = 3.56), *t*(64) = 3.27, *p* = 0.002, *d* = 0.41, or anonymous presentation of results (*M* = 1.12, *SD* = 3.43), *t*(64) = 2.64, *p* = 0.01, *d* = 0.33. However, participants in the present condition did not show the MD pull for either results presentation condition.

**FIGURE 2:**
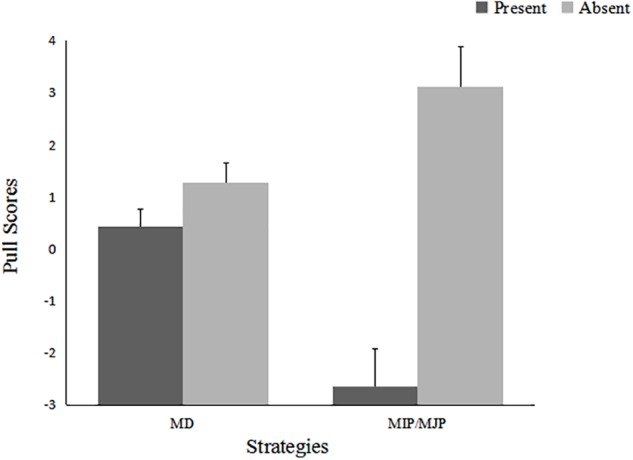
Pull scores as a function of competitive intragroup outcome interdependence and strategies: Experiment 1.

**FIGURE 3:**
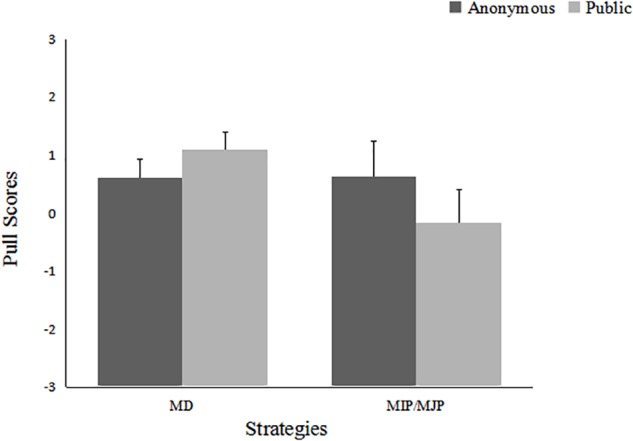
Pull scores as a function of results presentation and strategies: Experiment 1.

A 2 (Competitive Intragroup Outcome Interdependence) × 2 (Results Presentation) × 2 (Strategies: MD vs. MIP/MJP) ANOVA was conducted to determine the differing use of the above two strategies across the conditions. The analysis yielded a main effect of competitive intragroup outcome interdependence, *F*(1,137) = 29.27, *p* < 0.001, η^2^ = 0.176, and a significant Strategies × Competitive Intragroup Outcome Interdependence interaction, *F*(1,137) = 18.58, *p* < 0.001, η^2^ = 0.119, and a marginally significant Results Presentation × Strategies interaction, *F*(1,137) = 3.52, *p* = 0.063, η^2^ = 0.025. The means are presented in Figures 2, 3.

Further examination indicated that participants in the absent condition were more likely to exhibit MD and MIP/MJP strategies than were participants in the present condition, *F*(1,137) = 2.96, *p* = 0.088, η^2^ = 0.021; *F*(1,137) = 28.86, *p* < 0.001, η^2^ = 0.174. In the present condition, members’ negative MIP/MJP pull (*M* = -2.649, namely, maximizing outgroup profit/minimizing joint profit) was stronger than their MD pull (*M* = 0.432). In the absent condition, members’ positive MIP/MJP pull (*M* = 3.12) was stronger than their MD pull (*M* = 1.29). Simple effect tests for the Results Presentation × Strategies interaction indicated that the two pull scores did not differ in the anonymous presentation condition; however, in the public presentation condition, participants exhibited a stronger MD pull than an MIP/MJP pull, *F*(1,137) = 3.87, *p* = 0.05, η^2^ = 0.027.

### Discussion

Study 1 provides initial support for our prediction that ingroup favoritism would disappear when there is competition among ingroup members. More specifically, if the ingroup is in a situation where competitive intragroup outcome interdependence is not present, individuals tend to exhibit ingroup favoritism, regardless of whether their behavior is publicly broadcasted or anonymous. However, when competitive intragroup outcome interdependence is present, they would not exhibit ingroup favoritism behavior considering their own interests (again, regardless of the presentation of the results). Moreover, their strategies were consistent with their behavior: members demonstrated clear strategies of ingroup favoritism (a combination of MIP/MJP and MD) when competitive outcome interdependence was absent; but when it was present, they tended to show negative MIP/MJP strategy. Taken together, individuals’ intergroup behavior was based on their own interests. When their interests were threatened by ingroup members, they gave up favoring them.

Importantly, we did not consider social identity in Study 1. As noted above, SIT is a widely applied approach to explaining intergroup behavior in traditional social psychology (Tajfel and Turner, 1979; Turner et al., 1987). The degree of identification with a certain group seems to affect individuals’ perceptions and behaviors. People with higher identification are more likely to categorize themselves as ingroup members and remain faithful to the ingroup. Furthermore, greater group identification has been associated with increased helping behavior, trust, cooperation, and greater ingroup favoritism (Cremer and Stouten, 2003; Rand et al., 2009; Mcleish and Oxoby, 2011). Therefore, it seems necessary to consider the effect of social identity when exploring the relationship of competitive intragroup outcome interdependence and ingroup favoritism. In Study 2, we included social identity in the experiment employed in Study 1 to determine whether individuals’ degree of identification toward a group moderates their ingroup favoritism, especially under competitive intragroup outcome interdependence.

## Study 2

### Methods

#### Participants and Design

We employed a 2 (Competitive Intragroup Outcome Interdependence: present vs. absent) × 2 (Identification: high or low) between-subjects design. Sample size was determined, based on a between-subjects design ANOVA, by using G^∗^Power 3.1.9.2. With a hypothesized effect size = 0.25, α = 0.05, power = 0.8, four groups, and numerator df = 1, the estimated sample size was 128. To ensure robustness, we recruited 130 participants (85 female, 45 male; *M*_age_ = 21.04 years, *SD* = 1.65). They were randomly assigned to one of the two competitive intragroup outcome interdependence conditions (present: *n* = 68; absent: *n* = 62). Simultaneously, participants in each competitive intragroup outcome interdependence condition (present and absent) were averagely assigned to either high or low identification group by manipulating their identification. Thus, according to our design, four groups will be generated: present condition – high identification: *N* = 34; present condition – low identification: *N* = 34; absent condition – high identification: *N* = 31; absent condition – low identification: *N* = 31. After they completed the experiment, participants were given remuneration and thanked for their participation.

#### Procedure and Measures

The experimental procedure of Study 2 was similar to that in Study 1 except that it included a manipulation of identification grouping and an identification measure before participants were assigned to the present or absent competitive intragroup outcome interdependence conditions. Moreover, based on the results of Study 1 (i.e., no main effect of Results Presentation) and the suggestion of previous studies that individuals performed intergroup discrimination more truly behind the veil of anonymity, we conducted only the first round for Study 2 (i.e., the round of anonymous results presentation).

After categorizing participants into underestimator and overestimator groups, we began to manipulate their identification, referring to a method used by Leonardelli and Brewer (2001) and employed by Stroebe et al. (2005). We asked participants to complete a lifestyle questionnaire, in which they needed to rate whether each item of a list of ingroup characteristics was descriptive of themselves. This questionnaire included ten statements, each tailored to appear plausible as characteristics of “overestimators” or “underestimators” based on the participant’s categorization condition. Furthermore, each statement was preceded by a moderate frequency qualifier such as “sometimes” (e.g., “Sometimes, I find myself…”) or an extreme frequency qualifier “almost always” (e.g., “Almost always, I…”). According to Leonardelli and Brewer (2001), a statement preceded by moderate (or extreme) qualifiers will lead individuals to endorse (or reject) the statement as self-descriptive.

The experiment induced high and low identification by differing qualifiers in the same questionnaire. To induce high identification, eight out of ten descriptive statements were preceded by “sometimes,” with only two preceded by “almost always.” On the contrary, eight out of ten descriptive statements were preceded by “almost always,” with only two preceded by “sometimes” to induce low identification. All participants were asked to indicate whether each item was self-descriptive by circling the word “True” or “False.” Afterward, participants endorsed more statements (six or more), then read that they scored high on characteristics typical of category members, thereby inducing high identification with the ingroup. While participants who endorsed fewer statements (five or fewer) were led to believe that they scored low on characteristics typical of category members, thereby inducing weak identification with their group.

After the identification induction, participants were told to complete a social identification scale. They were then randomly assigned to the same one of the competitive intragroup outcome interdependence conditions, and were asked to complete tasks I and II, as in Study 1. The social identification scale developed by Leonardelli and Brewer (2001) was used to assess identification. This scale comprises 10 items rated on a 6-point Likert scale (1 = *strongly disagree*; 6 = strongly agree) and possible scores ranged from 10 to 60. Higher scores indicated stronger group identification. The internal consistency of the scale was acceptable (Cronbach’s α = 0.82).

### Results

#### Manipulation Checks

To effectively determine the effective of the manipulation for competitive intragroup outcome interdependence, the scores of perceived intragroup competition were submitted to a two-way ANOVA, with the identity manipulation and competitive intragroup outcome interdependence as between-participants factors. The analysis yield a significant main effect of competitive intragroup outcome interdependence on sense of intragroup competition, *F*(1,126) = 21.10, *p* < 0.001, η^2^ = 0.172. However, neither the main effect of identification nor the interaction effect reached significance. This indicated that, regardless of identification, participants in the present condition (*M* = 4.90, *SD* = 1.60) reported a higher sense of intragroup competition than did those in the absent condition (*M* = 3.42, *SD* = 1.67). Further, the data of perceived intragroup competition were submitted to one-sample *t*-tests. As expected, participants in the present condition indeed perceived a sense of competition with ingroup members (*M* = 4.89, *SD* = 1.60), *t*(67) = 4.61, *p* < 0.001, *d* = 0.56, while participants in the absent condition did not perceive any such sense (*M* = 3.42, *SD* = 1.68), *t*(61) = -2.73, *p* = 0.008, *d* = 0.35. Furthermore, a 2 (Competitive Intragroup Outcome Interdependence: present vs. absent) × 2 (Identification: high or low) between-subjects ANOVA on the scores of perceived reciprocal interdependence did not yield any main effect or interaction effect. The results for one-sample *t*-tests revealed that neither group of participants felt that they had depended on other participants for the reward [*M* = 3.02, *SD* = 1.70, *t*(67) = -4.79, *p* < 0.001, *d* = 0.58 and *M* = 2.55, *SD* = 1.34, *t*(61) = -8.54, *p* < 0.001, *d* = 1.08, respectively]. This indicated that we had successfully manipulated outcome interdependence within group and eliminated potential reciprocal interdependence among participants.

To effectively determine whether participants had been induced into high or low identification with their ingroup, we subjected the identification scale scores to a two-way ANOVA, with the identity manipulation and competitive intragroup outcome interdependence as between-participants factors. The analysis indicated a significant main effect of identity manipulation on identification, *F*(1,126) = 211.10, *p* < 0.001, η^2^ = 0.626. Participants in high identification manipulation reported significantly higher identification (*M* = 5.08, *SD* = 0.06) than did participants in low identification manipulation (*M* = 3.78, *SD* = 0.06). While, there were no significant main effect of competitive intragroup outcome interdependence and interaction effect. Namely, high induction manipulation increased identification for both present and absent conditions.

#### Ingroup Favoritism

The intra-class correlation coefficients were computed following the procedure suggested by Snijders and Bosker (1999), to confirm the independence of observations within groups. The results revealed if the targets were DS and MIP/MJP, the intra-class correlation coefficients were 0.32 and 0.1 respectively; if the target was MD, the intra-class correlation coefficient was trending to 0, indicating that the observations within groups were independent. Therefore, we then analyzed the data using the *t*-test and ANOVA.

A 2 (Competitive Intragroup Outcome Interdependence: present vs. absent) × 2 (Identification: high or low) between-subjects ANOVA on difference scores yielded a significant main effect of competitive intragroup outcome interdependence, *F*(1,126) = 28.07, *p* < 0.001, η^2^ = 0.182; this indicated that participants in the absent condition exhibited greater ingroup favoritism (*M* = 23.77, *SD* = 5.58) than did participants in the present condition (*M* = -17.10, *SD* = 5.33). However, neither the main effect of identification nor the interaction effect reached significance.

The mean difference scores of the participants in the absent condition were positive, whereas those of participants in the present condition were negative (as shown in Table 3). Using a series of one-sample *t* tests, we assessed participants’ intergroup bias through comparing their difference scores against zero for each group. In the present condition, the difference scores of participants with low identification was significantly lower than zero, *t*(33) = -2.89, *p* = 0.007, *d* = 0.50, while that of the high identification participants was marginally significantly lower, *t*(33) = -1.78, *p* = 0.085, *d* = 0.30; both results suggested outgroup favoritism. In contrast, in the absent condition, the difference scores of both high or low identification participants was greater than zero *t*(29) = 3.68, *p* = 0.001, *d* = 0.67; *t*(31) = 2.18, *p* = 0.037, *d* = 0.39. These results both indicated ingroup favoritism.

**Table 3:** Mean difference scores according to competitive outcome interdependence and group identification.

	Competitive outcome interdependence	
	Present	Absent
High identification	-13.44 (44.09)	30.23 (44.90)
Low identification	-20.77 (41.90)	17.31 (44.92)

*All values are Mean (SD)*.

#### Discrimination Strategies

**FIGURE 4:**
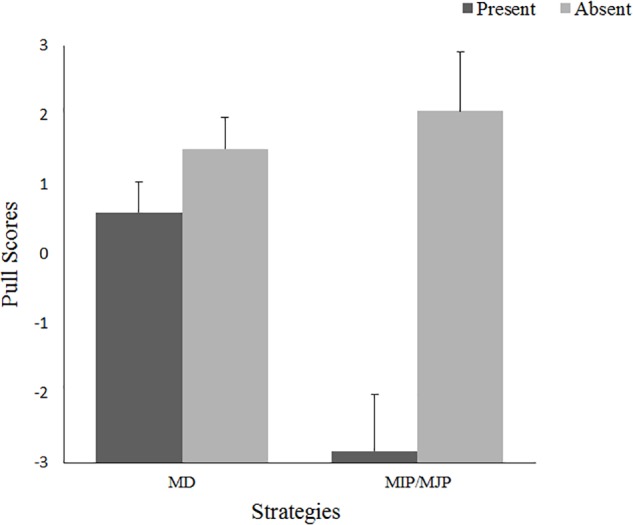
Pull scores as a function of competitive intragroup outcome interdependence and strategies: Experiment 2.

To determine the strategies of participants in the allocation task, we conducted several one-sample *t*-tests on the same pull scores assessed in Study 1. The results showed that in the absent condition, MD pull significantly differed from zero for participants with high identification, *t*(29) = 2.60, *p* = 0.014, *d* = 0.48, but only marginally, yet significantly, differed from zero for participants with low identification, *t*(31) = 1.79, *p* = 0.083, *d* = 0.32. For MIP/MJP pull, all participants, regardless of identification, exhibited a significantly negative MIP/MJP in the present condition, *t*(33) = -2.05, *p* = 0.049, *d* = 0.35, *t*(33) = -3.32, *p* = 0.002, *d* = 0.57; only participants with high identification exhibited a marginally significant MIP/MJP in the absent condition, *t*(29) = 1.86, *p* = 0.073, *d* = 0.34. Namely, participants had actually been used MD and MIP/MJP strategies. Next, we compared MD and MIP/MJP pulls across all of the conditions by subjecting them to repeated-measures ANOVAs, with competitive intragroup outcome interdependence and group identification as between-participants factors and the pull scores (strategies) as a two-level within-participants factor. The results indicated significant main effects of competitive intragroup outcome interdependence, *F*(1,126) = 18.78, *p* < 0.001, η^2^ = 0.13, and strategies, *F*(1,126) = 4.64, *p* = 0.033, η^2^ = 0.036. Furthermore, there was a significant interaction between competitive intragroup outcome interdependence and strategies, *F*(1,126) = 8.76, *p* = 0.004, η^2^ = 0.065. The means are presented in Figure 4.

In both the absent and the present conditions, members exhibited no difference in the MD pull score, but exhibited a significant difference in the MIP/MJP pull score, *F*(1,126) = 17.04, *p* < 0.001, η^2^ = 0.119. Participants in the present condition were more likely to exhibit negative MIP/MJP pull scores than were those in the absent condition. Furthermore, in the present condition, members’ negative MIP/MJP pull was stronger than their MD pull.

### Discussion

Study 2, wherein we considered group identification, replicated the results of Study 1; competition within groups indeed damaged ingroup favoritism. Specifically, in the present condition, participants did not exhibit a behavioral tendency to favor the ingroup and used a negative MIP/MJP strategy, regardless of their level of group identification. In contrast, in the absent condition, participants were more likely to exhibit ingroup favoritism. Interestingly, despite the apparent importance of identification in intergroup bias found in past studies, our results suggest that it has a relatively limited effect. Specifically, when competitive intragroup outcome interdependence was not perceived, we found that ingroup favoritism increased with identification. In contrast, when competitive intragroup outcome interdependence was perceived, identification could not moderate its destructive effect on ingroup favoritism.

## General Discussion

Individuals obtained a sense of security and psychological support from their ingroup, and tended to exhibit ingroup favoritism (Balliet et al., 2014). Ingroup competition may affect one’s behavior to his ingroup or ingroup members. We focused on whether intragroup competition would lead to the collapse of ingroup favoritism, and investigated how group identification affects the relationship between intragroup competition and ingroup favoritism. We used the minimal group paradigm across two experiments, demonstrating that competitive intragroup outcome interdependence indeed eliminates ingroup favoritism regardless of whether allocation results are public or anonymous; even group identification could not moderate its destructive effect.

BGR suggests that a basic principle of human behavior is to protect self-interest; therefore, people usually behave in a manner that maximizes self-gains. Accordingly, they are primarily concerned with whether their positive behaviors would yield reciprocity and help further their interests (Yamagishi et al., 1999). As the group provides benefits to its members, including acceptance, belongingness, social support, and a chance of repeated interactions among ingroup members, people hold a normative belief that ingroup members are more likely to reciprocate positive behaviors than outgroup members would (Balliet et al., 2014). This is called the “group heuristic.” Thus, BGR proposes that people favoring ingroup members are motivated by reciprocal expectations. When this “group heuristic” is not operational, individuals are not likely to favor ingroup members because the reciprocal expectation is absent (Rabbie et al., 1989; Velez, 2014). In the present study, we expected that the salience of competitive intragroup outcome interdependence would make individuals experience a conflict of interest with other ingroup members, thereby erasing reciprocal expectations. Thus, individuals did not exhibit ingroup favoritism in order to avoid the potential loss of their own benefit.

Ingroup favoritism is a way of developing and maintaining a positive self-concept for those who identify with a certain group. People feel that their interests are aligned with the interests of the group, and therefore help group members just as they help themselves because of group identification (Everett et al., 2015). This implies that identification is substantially related to individuals’ motivation to pursue a positive self-concept and self-interests. In the context of the absence of internal competition, the effect of identification on one’s behavior was consistent with the effect of pursuing individual benefits. Consequently, people exhibited ingroup favoritism, and the higher identification, the stronger ingroup favoritism they exhibited (Cremer and Vugt, 2002). Nevertheless, SIT tends to assume that individuals view their social group in a positive light and expect positive mutual interdependence among ingroup members (Beauchamp and Dunlop, 2014). Moreover, group identification does not automatically lead to favoring of the ingroup over the outgroup (Ellemers and Haslam, 2011). In the present study, when exposed to an environment of intragroup competition, individuals found it difficult to benefit from the group, so their identification and motivation of pursuing positive self-concept and benefits clashed; thus, the collapse of ingroup favoritism could not be prevented.

Self-categorization theory (SCT) offers an explanation of why individuals’ group identification does not moderate the destructive effect of internal competition on ingroup favoritism. Based on SCT, there are three levels of self-categorization that are important to one’s self-concept: human identity, social identity, and personal identity, which vary depending on its salience in the context, where one level becomes more salient than the others through a process of categorization (Hornsey, 2008). The process occurs through two primary mechanisms: fit and accessibility (Oakes, 1987; Oakes et al., 1991). One of the components of fit—normative fit—represents the extent to which people’s behaviors in certain social categories align with stereotypical expectations. If one’s behavior is inconsistent with what might be expected from members of that group, this may reduce the salience of the group identity (Beauchamp and Dunlop, 2014). In the present study, individuals may have assumed that other members would not exhibit the anticipative group norm of favoring the ingroup in the competitive context, which would thus reduce the salience of their group identity. Simultaneously, accessibility (also called “perceived readiness”) is heavily dependent on a person’s current goals and context. Categories are more likely to be accessible if they are accessible in the moment or are primed for the situation (Hornsey, 2008; Beauchamp and Dunlop, 2014). When perceiving competitive intragroup outcome interdependence, personal identity rather than group identity becomes more accessible for protecting one’s own interest. This salience of personal identity would in turn lead to the pursuit of personal interests and thus abandonment of ingroup favoritism.

Another speculation on the moderation deficit of identification is that intragroup competition not only reduced the salience of group identity, but also directly impaired one’s group identification. Thus, while the weakened identification could not alter the destructive effect of competitive intragroup outcome interdependence on ingroup favoritism, it relatively increased ingroup favoritism in the context of absent competitive intragroup outcome interdependence. A previous study claimed outgroup threat or competition generated hostility toward the outgroup, which could increase ingroup identification and cohesion (LeVine and Campbell, 1972). Therefore, it is reasonable to suppose that intragroup competition could decrease group identification. Moreover, Realistic Conflict Theory assumes that positive relations can only be achieved if superordinate goals are in place and conflict occurs when there is competition for scarce resources (Mutezo, 2015). Negative relations and feelings are always accompanied by conflict or competition for scarce resources. However, negative feelings about the ingroup, such as fear and uneasiness, would motivate a decrease in identification (Muldoon et al., 2010). Under competitive intragroup outcome interdependence, a competitive relationship with ingroup members might cause individuals to generate negative feelings directed at the ingroup, and in turn, weaken their group identification. Thus, competitive intragroup outcome interdependence leads to the collapse of ingroup favoritism through weakening identification. However, the present study neither aimed to explore why intragroup competition damaged ingroup favoritism nor to reveal the mechanism of how psychological distance destroys ingroup favoritism. Therefore, we did not measure group identification after the manipulation of competitive intragroup outcome interdependence, and consequently, we could not confirm this speculation. Future research should focus on this mechanism.

Although competitive intragroup outcome interdependence was found to change the tendency of ingroup favoritism (by plausibly making participants exhibit outgroup favoritism), the analysis of strategies revealed some interesting details about that tendency. Specifically, in the absent condition, participants employed MD and MIP strategies when favoring ingroup members; while in the present condition, they only preferred to maximize outgroup profit rather than maximize differences in favor of the outgroup. Combining the traits of Tajfel’s allocation matrices, individuals allocated fewer points to ingroup members such that outgroup members could get more points. In the current study, the fewer points individuals allocated to ingroup members, the greater was the likelihood that they would gain reward. Thus, plausible outgroup-favoring behavior is merely an appendant manifestation of the pursuit of one’s own interests rather than an authentic preference for the outgroup.

In conclusion, we used the classic Tajfel minimal group paradigm matrices and relied on effective manipulation of outcome dependence to elucidate the destructive effect of competitive intragroup outcome interdependence on ingroup favoritism. Combining the analysis of SIT and BGR, our results contribute to a better understanding of ingroup favoritism. Namely, intragroup competition eliminates ingroup favoritism by erasing reciprocal expectations toward ingroup members and influencing the process of self-categorization, thus inhibiting the influence of group identification. The research cautions the importance of focusing on the negative effect of competitive intragroup outcome interdependence on ingroup favoritism. Future research needs to find a way to buffer this negative effect to facilitate internal harmoniousness and the development of group.

There are some limitations of the present study that must be acknowledged. First, participants in the minimal group paradigm engage in a transitory connection, which means that even for those who reported high identification, there might have been a higher degree of perceived social distance between participants. Simultaneously, considering we only employed artificial groups in the present study, it is possible that among groups with a shared culture and history, ingroup competition does not have such dramatic effects. Future research could benefit from exploring whether the negative effect of competitive outcome interdependence on ingroup favoritism would emerge in real groups. Furthermore, participants may have been strongly motivated to pursue the additional reward because it was equal to the fundamental remuneration. It would be interesting to explore whether varying the rate of the reward (to half or less) would change participants’ discriminatory behavior. Moreover, we did not find a significant effect of result presentation. It is possible that manipulating the form of the allocation might make participants think more about their reputation, and thus adopt varying allocation strategies. Finally, although we elucidated the influence of competitive intragroup outcome interdependence on ingroup-favoring behavior via an economic game, future research needs to further verify its influence of on cognition and affect.

## Author Contributions

YfZ substantial contributions to revise the paper critically for important intellectual content. YxZ completing the whole study and be responsible for writing the paper. BC giving a great deal of help during the acquisition, analysis and interpretation of data. YfZ, YxZ, and BC final approval of the version to be published, and got agreement to be accountable for all aspects of the work in ensuring that questions related to the accuracy or integrity of any part of the work are appropriately investigated and resolved.

## Conflict of Interest Statement

The authors declare that the research was conducted in the absence of any commercial or financial relationships that could be construed as a potential conflict of interest.

## Supplementary Material

DATA SHEET S1 Data from study 1.

DATA SHEET S2 Data from study 2.
